# P53 Alleviates the Progression of Periodontitis by Reducing M1-type Macrophage Differentiation

**DOI:** 10.1007/s10753-024-01968-w

**Published:** 2024-02-06

**Authors:** Tingting Liu, Dongru Chen, Shanshan Tang, Zhaolei Zou, Fangyi Yang, Yutian Zhang, Dikan Wang, Huanzi Lu, Guiqing Liao, Xiangqi Liu

**Affiliations:** 1 Hospital of Stomatology, Guanghua School of Stomatology, Sun Yat-Sen University, Guangdong Provincial Key Laboratory of Stomatology, Guangzhou, China; 2 Department of Oral and Maxillofacial Surgery, Hospital of Stomatology, Sun Yat-Sen University, Guangzhou, China

**Keywords:** macrophage, P53, inflammation, periodontitis, LPS

## Abstract

Our objective is to explore the effect of P53 on the progression of periodontitis by regulating macrophages differentiation both *in vitro* and *in vivo.* Eighteen normal and periodontitis gingival tissues were collected for detecting P53 expression and macrophages infiltration by immunofluorescence, real-time PCR (qPCR) and western-blot. The differentiation and the inflammatory cytokines (TNF-α and IL-6) expression of THP-1, RAW264.7 and bone marrow derived macrophage (BMDM) cells, treating with Pifithrin-α (P53 inhibitor) or Nutlin-3a (P53 activator) under lipopolysaccharide (LPS) stimulation, were observed by flow cytometry, qPCR and ELISA. The severity of periodontitis, inflammatory cytokines expression and macrophages infiltration were measured in experimental periodontitis wild-type mice and *p53* gene conditional knocked-out (*p53*-CKO) mice, which were established by ligation and LPS injection. A higher number of P53-positive macrophages was found infiltrated in periodontitis tissues. *In vitro* experiments showed that compared with Nutlin-3a, the proportion of M1-type macrophages and the expression of TNF-α and IL-6 were higher in Pifithrin-α treated cells under LPS stimulation. *In vivo* experimental periodontitis mice, the Pifithrin-α intraperitoneal injection group showed greater alveolar bone loss, higher levels of TNF-α and IL-6 secretion and more M1-type macrophages infiltration, while the Nutlin-3a intraperitoneal injection group were observed mild symptoms compared with mice in the periodontitis group. *P53*-CKO mice exhibited more severe periodontitis and more M1-type macrophages infiltrated in local tissues compared with wild-type mice. The activation of *p53* gene could alleviate periodontitis by reducing M1-type macrophage polarization. P53 may serve as keeper in the progression of periodontitis, providing new insights into periodontitis treatment.

**Supplementary Information:**

The online version contains supplementary material available at 10.1007/s10753-024-01968-w.

## INTRODUCTION

Periodontitis is triggered by the interaction between the dysregulated microbial community and the abnormal immune response within the gingival and periodontal tissues [1, 2]. Not only does periodontitis damage local periodontal tissue, but it also triggers distant organ diseases such as inflammatory bowel disease [3], arthritis [4], cirrhosis of the liver [5] and even colorectal cancer [6]. Some immune related genes, such as the genetic modification of the Purinergic receptor P2X7 (P2X7R) [7], Tumor Necrosis Factor receptor-immunoglobulin Fc (TNFR: Fc) [8], Toll-like receptor 2 (TLR2) and Myomesin 2 (MYOM2) genes [9] are of great significance for the progression of periodontitis, and regulating the expression of related genes may be useful for the treatment of periodontitis.

P53 has a broad range of effects on inflammation [10]. Evidences have shown that the increased expression of *p53* gene is related to chronic renal inflammation [11, 12] and multiple peripheral artery occlusions [13]. However, other studies found that increased P53 expression could improve acute liver injury [13, 14]. In addition, the lack of P53 function contributes to the pathogenesis of rheumatoid arthritis [15]. For periodontitis, most studies focused on the P53 expression level of gingival fibroblasts. Some researchers found higher P53 expression on periodontitis than healthy tissues [16], but others found no changes between them [17]. Moroever, periodontitis also consists of immune cells, whether P53 affects the progression of periodontitis by immune cells is still unclear.

Macrophages play a key role in both the destructive and reparative phases of periodontal disease [18, 19], as they differentiate into M1-type during the early stages of periodontitis and secrete high levels of pro-inflammatory cytokines such as IL-1β, IL-6, TNF-α, and IFN-γ [20, 21]. As the disease progresses into its restorative phase, the proportion of M2-type macrophages increases, and they secrete more anti-inflammatory cytokines such as IL-10. In some inflammatory diseases, P53 has been shown to have a regulatory effect on macrophages. For example, P53 activation has been shown to relieve tuberculosis by stimulating macrophages polarization to M1-type [22]. While P53 inhibition has been found to promote macrophage polarization to M2-type in sepsis-induced lung injury [23]. Based on these findings, we hypothesize that P53 may affect macrophage polarization and inflammatory cytokines secretion in periodontal disease.

At present, the role of P53 regulating macrophage differentiation in periodontitis is still not very clear. Therefore, this study aims to explore the effect of P53 on the progression of periodontitis by regulating macrophages differentiation both *in vitro* and *in vivo*. We hope that the findings of this study will provide new perspectives and contribute to the development of periodontitis treatment in the future.

## MATERIAL AND METHODS

### Human Gingival Tissue

Nine normal gingival tissue samples and nine periodontitis tissue samples were collected from Hospital of Stomatology, Sun Yat-sen University. Normal samples were taken from patients with non-inflamed periodontal tissue after impacted tooth extraction. Periodontitis samples were obtained from patients with severe periodontitis, which the tooth could not be retained. This study was approved by The Ethics Committee of Hospital of Stomatology, Sun Yat-sen University. And this study conforms to recognized standard of Declaration of Helsinki.

### Cell Preparation and Acquisition

THP-1 and RAW264.7 cells were obtained from the Cell Bank of the Chinese Academy of Sciences (Shanghai, China). The THP-1 cell line is derived from leukemia monocytes which can be stimulated to differentiate into macrophage cells. RAW 264.7 cells are a macrophage-like, Abelson leukemia virus-transformed cell line derived from BALB/c mice. They were cultured in Roswell Park Memorial Institute (RPMI) 1640 medium (Gibco, USA) and Dulbecco's modified Eagle's medium (DMEM, Biosharp, China), respectively. The medium was supplemented with 1% penicillin-streptomycin (Gibco, USA) and 10% fetal bovine serum (FBS, Gibco, USA). Bone marrow cells were isolated from the tibias and femurs of C57 mice and cultured in RPMI 1640 medium containing 10% FBS, 1% penicillin-streptomycin, and 35 ng/mL recombinant M-CSF (Abbkine, USA) for 5 days to obtain bone marrow derived macrophage (BMDM) cells.

### Mice

Six‐week‐old male C57BL/6 mice were purchased from GemPharmatech (Nanjing, China). The C57BL/6 background *p53*^flox/flox^; Lyz2-Cre conditional knockout (*p53*-CKO) mice, which conditioned knockout p53 gene of myeloid cells, were produced from Shanghai Model Organisms Center, Inc. (China). The mice were housed in a specific pathogen-free facility, and all animal procedures were conducted in compliance with the guidelines of the Institutional Review Board (IRB) and Institutional Animal Care and Use Committee (IACUC) of Sun Yat-Sen University (Approval No. SYSU-IACUC-2023-000246).

Wild-type or *p53*-CKO mice were used to construct periodontitis models by ligating bilateral maxillary second molars with 5-0 silk ligature, and injecting 5ul *porphyromonas gingivalis* LPS (*Pg*.LPS, 1 mg/ml) into gingival sulcus on the buccal and lingual sides 3 times a week for 10 days.

### Real-time Polymerase Chain Reaction (qPCR)

RNA extraction was carried out with RNA-Quick Purification Kit (YiShan Biotech, China). RNA concentrations and purity were measured using a Nanodrop-1000 spectrophotometer (Thermo Fisher Scientific, US). Total RNA (1000 ng) was reverse transcribed into cDNA using the PrimeScript^™^ RT reagent Kit (Takara, Beijing). Then the resulting cDNA was used for subsequent analyses or stored at -80 °C until further use. qPCR was performed on a LightCycler 480 system (Roche, US). Primers used for qPCR were listed as Supplementary Table.

### Western Blot (WB)

The total proteins were extracted from cultured cells, human gingival tissue, or mouse periodontal tissue using radioimmunoprecipitation assay (RIPA) buffer (Solarbio, China). The concentration of protein was determined by BCA Protein Assay Kit (CWBIO, Beijing, China). Protein samples (20 µg) were separated by protein preformed gel (ACE Biotechnology, China) and transferred onto a polyvinylidene difluoride (PVDF) membrane (Millipore, USA). The membrane was incubated with anti-p-P53/P53 (Cell Signaling Technology, USA) overnight, and the expression of p-P53/P53 was detected using chemiluminescence (Millipore, USA).

### Immunofluorescence (IF)

The sections of mice periodontal tissue were incubated with F4/80 (Thermo Fisher Scientific, America, 1:300) and CD86 (Cell Signaling Technology, USA, 1:300) or CD206 (HUABio, China, 1:300) at 4 °C overnight. The sections of human gingiva were incubated with CD68 (Cell Signaling Technology, USA, 1:300) and P53 (Cell Signaling Technology, USA, 1:2000) at 4 °C overnight. Then, the Alexa Fluor 488 Goat Anti-Rabbit IgG (Beijing Emarbio Science &Technology Company, China, 1:500) and Alexa Fluor 594 Goat Anti-Mouse IgG (Beijing Emarbio Science &Technology Company, China, 1:500) were added and incubated for 1 h in the dark. DAPI (Beyotime, Shanghai, China) was used to stain the slides for 5 min. Finally, the samples were observed under a fluorescence microscope (Olympus Corporation, Tokyo, Japan).

### Flow Cytometry

The cultured cells were incubated with 0.125 µg of allophycocyanin (APC)-labeled CD86 (eBiosciences, USA) or allophycocyanin (PE)-labeled CD86 (Bioledgend, USA) for 30 min. After that, the cells were fixed with IC Fixation Buffer (Thermo Scientific, USA), and then incubated with 0.25 µg of Fluorescein Isothiocyanate (FITC)-labeled CD206 (BioLegend, USA) on ice for 30 min. Finally, flow cytometric analyses were performed using BD LSRFortessa (BD Biosciences, USA).

### Enzyme-linked Immunosorbent Assay (ELISA)

ELISA kits were used to measure the levels of IL-6 and TNF-α in cell culture supernatants and mice serum according to the manufacturer's instructions (MultiSciences (Lianke) Biotech Co., Ltd., China). Serum was obtained by resting the intravenous blood sample at room temperature for 30 min, then centrifuging it at 1500 g for 10 min in a refrigerated centrifuge.

### Hematoxylin–eosin Staining (HE)

Mice maxillae were decalcified in a slow EDTA decalcification solution (Wuhan Servicebio, China) for 1 month, with the solution being changed every 2 days. Human gingiva samples were fixed in 4% paraformaldehyde (Beijing Solarbio Science & Technology Co.,Ltd., China) for 24 h after dehydration, embedded in paraffin, and sliced into 5 μm sections for hematoxylin and eosin (HE) staining. The sections were then observed under a light microscope.

### Micro-computed Tomography (CT) Analysis

One side of the maxillary jaws was scanned using micro-CT (Scano Medical AG, Switzerland) to assess alveolar bone loss and bone density. The X-ray source was set as follows: 70 kV and 200 μA and resolution 10 μm. The three-dimensional images were reconstructed using Mimics Research 21.0 (Materialise, Belgium). The distance between the cementoenamel junction and the alveolar bone crest (CEJ-ABC) was measured on the mesial root surface of the second molars to evaluate the level of bone absorption. A longer distance from CEJ to ABC suggests more bone loss. The bone histomorphometric parameters bone volume/tissue volume ratio (BV/TV) was determined using CT-Analyzer software.

### Statistical Analysis

The statistical analysis was performed using GraphPad Prism 9.0.0 (San Diego, CA, USA). The data were analyzed using a two-tailed t-test, one-way ANOVA and multiple comparisons test. Punctuations indicate significant differences between experimental groups (*p < 0.05, **p < 0.01, ***p < 0.001, ****p < 0.0001).

## RESULTS

### P53 Expression was Elevated in Human Periodontitis Gingival Tissue and Macrophages

**Fig. 1:**
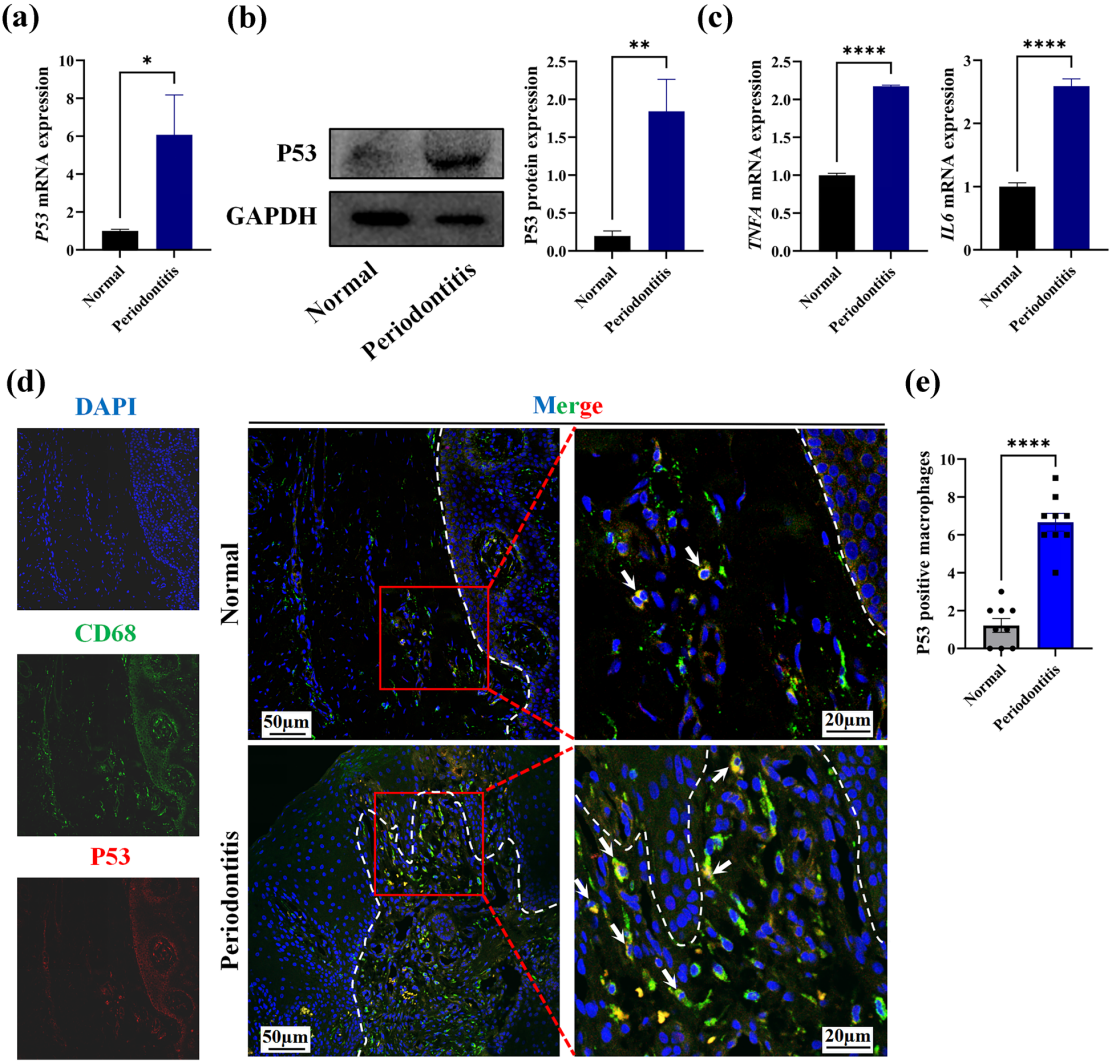
High expression level of inflammatory cytokines and increasing P53-positive macrophages were detected in periodontitis tissues. **a** The relative expression level of *P53* mRNA in normal and periodontitis tissues. **b** The expression of the P53 protein in normal and periodontitis tissues. **c** The gene expression level of TNF-α and IL-6 in normal and periodontitis tissues. **d** The representative immunofluorescence picture of P53-positive macrophages infiltrated in normal and periodontitis tissues. Blue: DAPI, Green: CD68, Red: P53. The white arrows indicated P53-positive macrophages. **e** The number of P53-positive macrophages infiltrated in normal and periodontitis tissues (*n* = 9). *p < 0.05, **p < 0.01, ****p < 0.0001.

We firstly examined the gene and protein expression levels of P53 in human gingival tissue. Compared to the healthy control group, our results verified that the *P53* gene and P53 protein expression levels were elevated in the periodontitis group (Fig. 1a, b). Moreover, we also observed that pro-inflammatory cytokines TNF-α and IL-6 had higher gene expression in the periodontitis group (Fig. 1c).

Next, we investigated P53 expression on macrophages in healthy gingival tissue and periodontitis gingival tissue. Our findings showed that the expression of P53-positive macrophages was significantly higher in periodontitis tissue (Fig. 1d, e). Based on these results, we hypothesize that P53 expression on macrophages may play a role in controlling periodontal inflammation.

### Regulation of P53 does not Influence Macrophage Differentiation in the Resting State

**Fig. 2:**
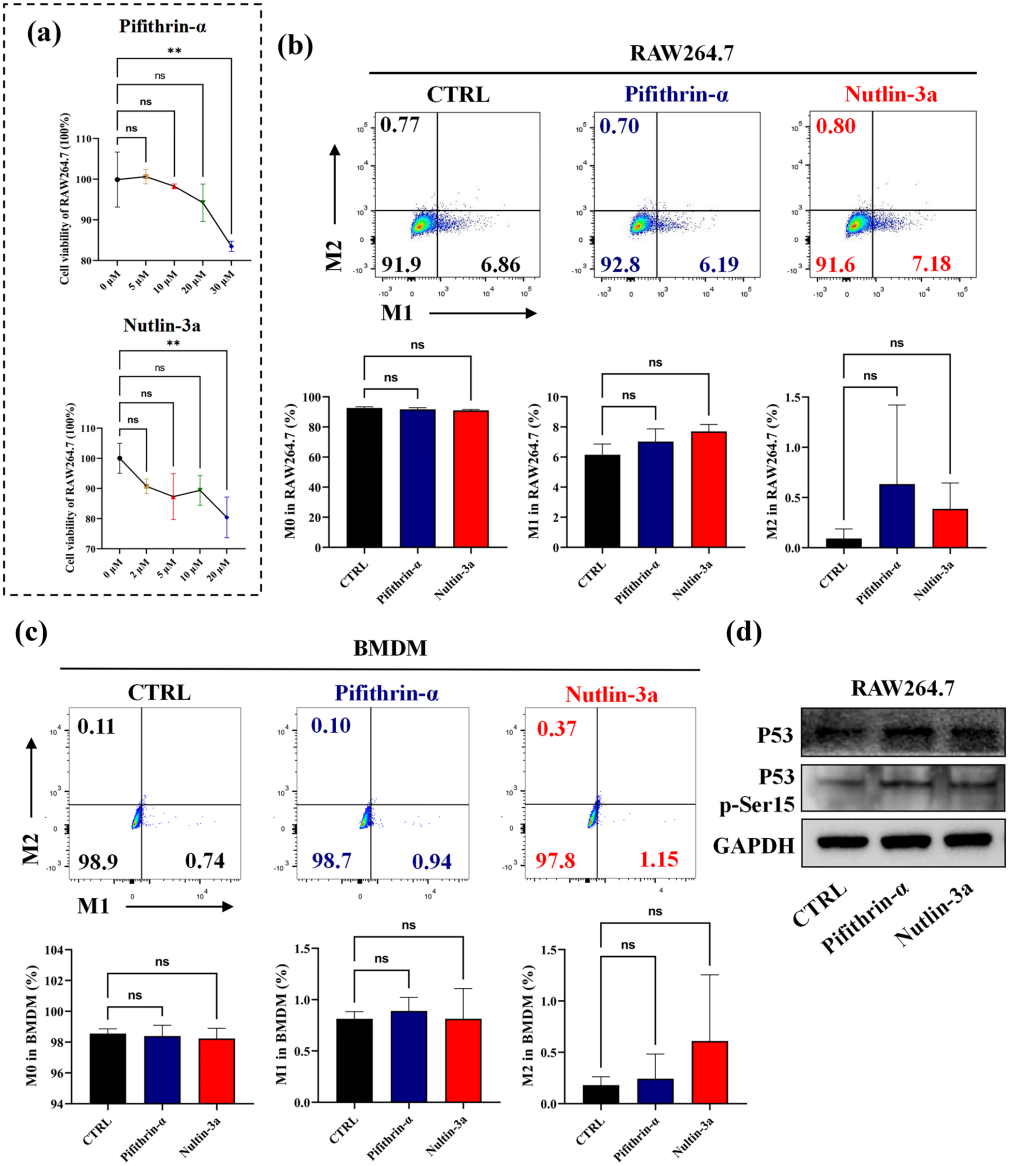
P53 has no significant effect on macrophage differentiation without any stimuli. **a** The cell viability of RAW264.7 cells (4 × 10^5^ cells/well) was detected by CCK8 after co-culture with different concentration of Pifithrin-α and Nutlin-3a for 24 h. **b**–**d** Loading RAW264.7 (4 × 10^5^ cells/well) and BMDM (6 × 10^5^ cells/well) in a six-wells plate for 24 h, then the cells were cultured in the presence of 20 μM Pifithrin-α or 10 μM Nutlin-3a for 24 h. Next, the cells were harvested for flow cytometry or western-blot. **b** The proportion of M0, M1 (CD86) and M2 (CD206) macrophages in RAW 264.7 cells. **c** The proportion of M0, M1 and M2 macrophages in BMDM cells. **d** The expression of total P53 protein and phosphorylation P53 protein in RAW 264.7 cells. ns: no significant, **p < 0.01.

To investigate the role of P53 regulation on macrophage differentiation, we firstly wanted to know whether regulation of P53 could affect macrophage differentiation in the absence of other stimuli. We screened and found the most suitable concentrations of Pifithrin-α (P53 inhibitor) and Nutlin-3a (P53 activator) for this experiment were 20 μM and 10 μM, respectively (Fig. 2a).

Our results showed that the application of Pifithrin-α or Nutlin-3a had no effect on the differentiation to M1 or M2 macrophage of THP-1 (Supplementary Fig. 1A). Similarly, we further validated no obvious effect on the differentiation to M1 or M2 macrophage with the application of Pifithrin-α or Nutlin-3a in murine cell line RAW264.7 and mice BMDM (Fig. 2b, c). The BMDM morphology was observed and the induction efficacy of BMDM cells was above 90% (Supplementary Fig. 1B, C). Additionally, the total P53 protein and P53 p-Ser15 protein expression also showed no significant change in RAW264.7 cells with the application of Pifithrin-α or Nutlin-3a (Fig. 2d). Based on these results, we speculate that the inhibition or activation of P53 do not have a significant effect on macrophage differentiation in the resting state.

### P53 Inhibits Macrophage Polarizing to M1-type Under *porphyromonas gingivalis* LPS Stimulation

Since periodontitis is chronic inflammatory disease, we further selected LPS from *porphyromonas gingivalis* (*Pg*.LPS) to activate inflammation. We firstly stimulated THP-1, RAW264.7 and BMDM cells with LPS from *porphyromonas gingivalis* (*Pg*.LPS) and/or IFN-γ, confirming that the polarization to M1-type macrophage was successful (Supplementary Fig. 1D–F). The cell morphology of the LPS+Pifithrin-α group exhibited more differentiated cells, while the LPS+Nutlin-3a group showed less differentiated cells (Supplementary Fig. 2A).

**Fig. 3:**
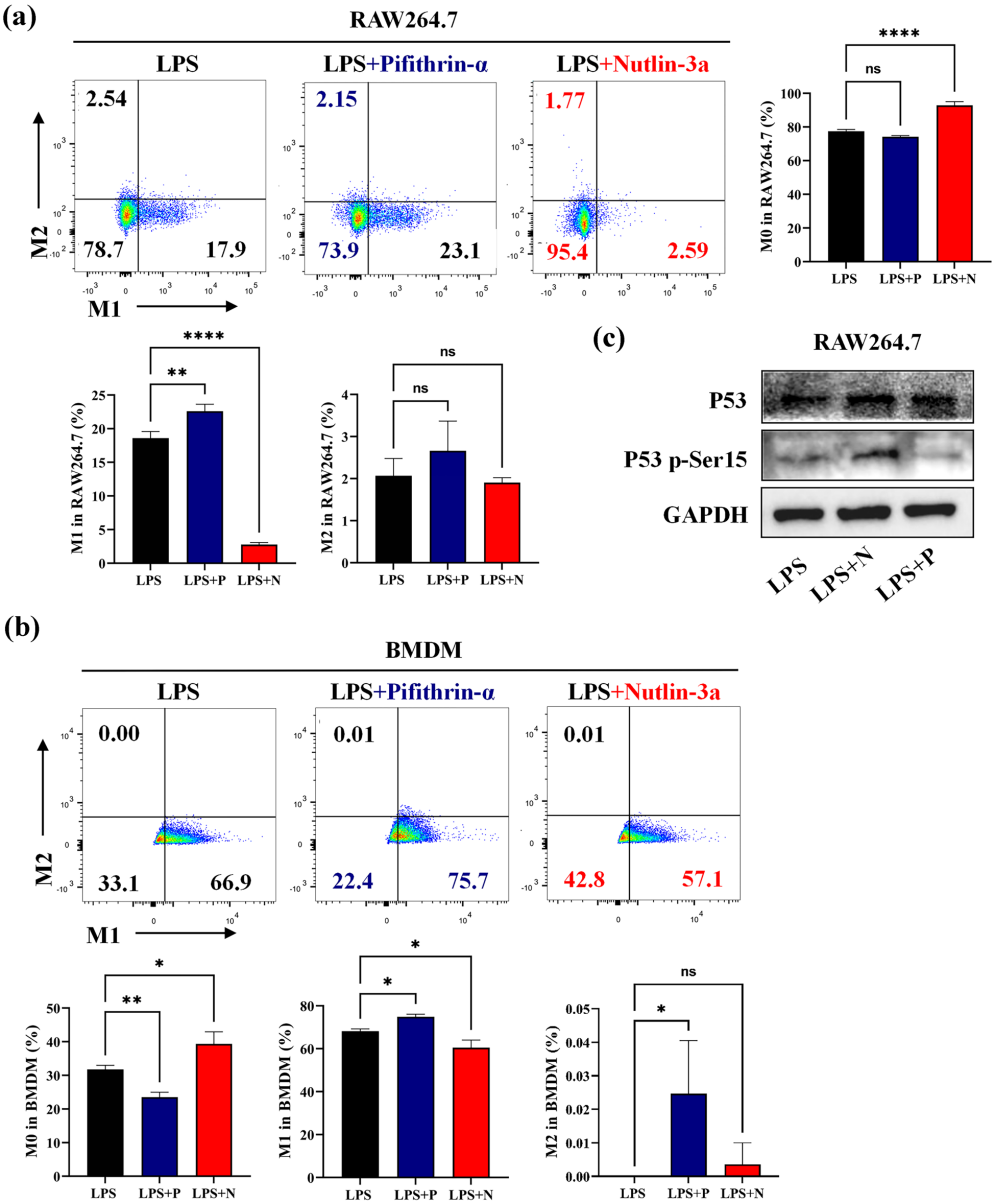
Inhibition P53 activity can promote macrophage polarize to M1-type under *porphyromonas gingivalis* LPS (*Pg.*LPS) stimulation RAW264.7 (4 × 10^5^ cells/well) and BMDM (6 × 10^5^ cells/well) cells co-cultured with 20 μM Pifithrin-α or 10 μM Nutlin-3a in a six-wells plate for 24 h, then the cells were exposed with *Pg.*LPS (1 µg /mL) for another 24 h. Next, the cells were harvested for flow cytometry or western-blot. **a** The proportion of M0, M1 (CD86) and M2 (CD206) macrophages in RAW 264.7 cells. **b** The expression of total P53 protein and phosphorylation P53 protein in RAW 264.7 cells. **c** The proportion of M0, M1 and M2 macrophages in BMDM cells. LPS: lipopolysaccharide, P: Pifithrin-α, N: Nutlin-3a. ns: no significant, *p < 0.05, **p < 0.01, ****p < 0.0001.

Compared to the LPS group, results showed that the proportion of M1 in THP-1, RAW264.7 and BMDM cells increased significantly after the addition of Pifithrin-α, and decreased markedly with Nutlin-3a (Supplementary Fig. 2B, Fig. 3a, b). Meanwhile, the P53 p-Ser15 protein expression decreased in the LPS+Pifithrin-α group and increased in the LPS+Nutlin-3a group (Fig. 3c). However, no obvious change was found in the proportion of M2 macrophages (Supplementary Fig. 2B, Fig. 3a, b). Above results showed the regulation of P53 activity could affect M1-type macrophage differentiation under *Pg.*LPS stimulation.

### Inhibiting P53 activation can increase the inflammatory cytokine expression in *Pg*.LPS-induced macrophage

**Fig. 4:**
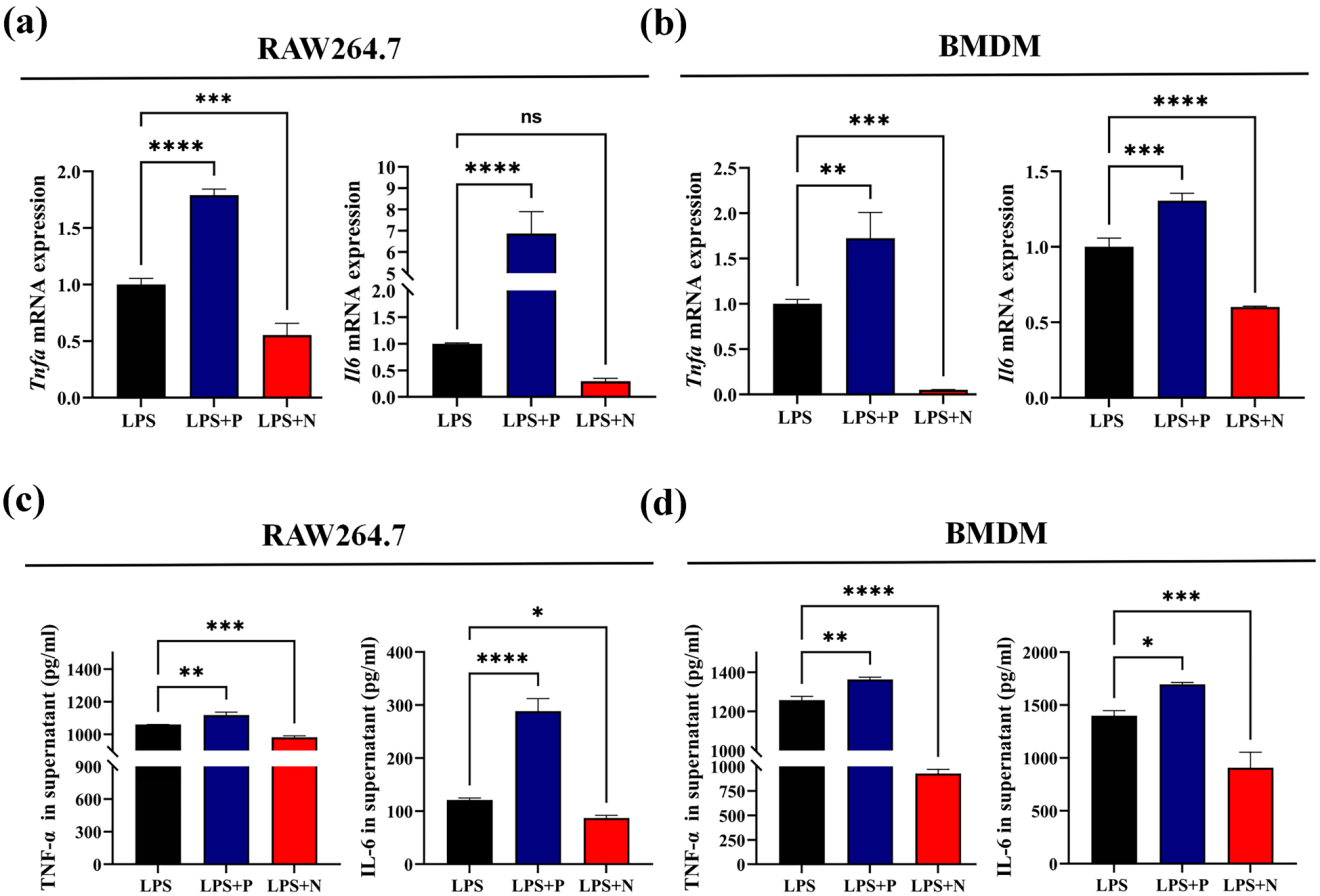
Suppressing P53 function can increase the expression of inflammatory cytokines in *Pg.*LPS-induced macrophage The *Pg.*LPS-induced RAW264.7 or BMDM cells were harvested for real-time PCR (qPCR), and the cultured supernatant were collected for ELISA. **a** The expression of *Tnfa* and *Il6* in RAW264.7 cells. **b** The expression of *Tnfa* and Il6 in BMDM cells. **c** The secretion of TNF-α and IL-6 in cultured supernatant of RAW264.7 cells. **d** The secretion of TNF-α and IL-6 in cultured supernatant of BMDM cells. LPS: lipopolysaccharide, P: Pifithrin-α, N: Nutlin-3a. ns: no significant, *p < 0.05, **p < 0.01, ***p < 0.001, ****p < 0.0001.

To further understand the effects of P53 regulation on macrophage polarization, we firstly detected the expression and secretion of inflammatory cytokines TNF-α and IL-6 were significantly increased in *Pg.*LPS-induced macrophages (Supplementary Fig. 2C, D). Next, to determine whether these cytokines from *Pg.*LPS-induced macrophages could change in pretreating with Pifithrin-α or Nutlin-3a, results showed the mRNA expression of TNF-α and IL-6 were significantly increased in both RAW264.7 and BMDM cells with the addition of Pifithrin-α, and decreased with Nutlin-3a (Fig. 4a, b). Meanwhile, the concentrations of TNF-α and IL-6 in the cell cultured supernatant were also increased in the group pretreated with Pifithrin-α and decreased in the group pretreated with Nutlin-3a (Fig. 4c, d). These results verified that changing P53 activity could regulate the expression of inflammatory cytokines of *Pg.*LPS-induced macrophages. Therefore, we speculate that regulating P53 activity could affect the inflammatory progression by inducing macrophage differentiation.

### P53 Inhibitor Pifithrin-α Accelerates the Periodontitis Severity by Promoting the M1-type Macrophage Infiltration

**Fig. 5:**
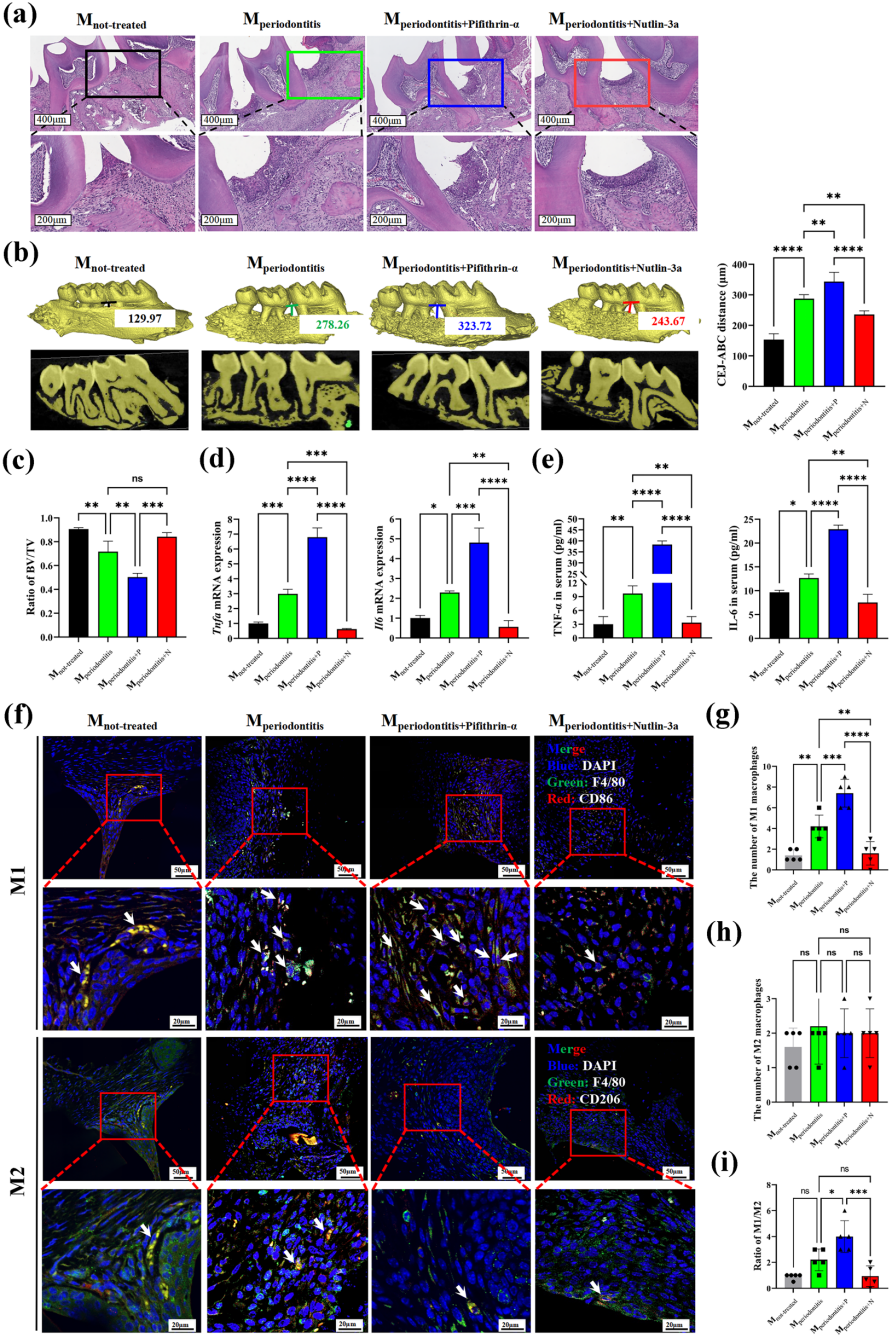
Pifithrin-α can accelerate the periodontitis severity by promoting the M1-type macrophage infiltration in experimental periodontitis mice Not-treated group (M_not-treated_, *n* = 5) was only injected with physiological saline intraperitoneally every 2 days. Periodontitis group (M_periodontitis_, *n* = 5) was injected with physiological saline intraperitoneally. Periodontitis + Pifithrin-α group (M_periodontitis+P_, *n* = 5) or Periodontitis + Nutlin-3a group (M_periodontitis+N_, *n* = 5) were injected with Pifithrin-α solution (20 mg/kg) or Nutlin-3a solution (10 mg/kg) intraperitoneally every 2 days. **a** The representative HE staining picture of mice periodontitis tissues in each group. **b** The absorption degree of alveolar bone in each group. Representative micro-CT picture was in the left, and the statistics was in the right. **c** The bone mass changes of alveolar bone in each group. **d** The mRNA expression of TNF-α and IL-6 in mice periodontal tissues (*n* = 3). **e** The concentration of TNF-α and IL-6 in serum of mice (*n* = 3). **f** The representative immunofluorescence picture of M1-type and M2-type macrophages infiltrated in mice periodontal tissues. For M1-type macrophages, Blue: DAPI, Green: F4/80, Red: CD86. For M2-type macrophages, Blue: DAPI, Green: F4/80, Red: CD206. The white arrows indicated M1-type or M2-type macrophages. **g**, **h** The number of M1-type macrophages (**g**) and M2-type macrophages (**h**) infiltrated in each group of mice periodontal tissues. **i** The ratio of M1-type/M2-type macrophages in each group. BV: bone volume, TV: tissue volume, CEJ-ABC: Cemento enamel junction-Alveolar bone crest, P: Pifithrin-α, N: Nutlin-3a. ns: no significant, *p < 0.05, **p < 0.01, ***p < 0.001, ****p < 0.0001.

Firstly, the periodontal tissue was observed using HE staining after inducing periodontitis in mice, showing increased infiltration of inflammatory cells, pronounced prolongation of epithelial pegs, and proliferation of bound epithelium towards the root (Supplementary Fig. 3A). The experimental methods are shown in Supplementary Fig. 3B. Mice in Pifithrin-α group showed worse periodontitis, while mice in Nutlin-3a group exhibited relatively relieved periodontitis (Fig. 5a). To assess alveolar bone resorption and bone mass, Micro CT was used to detect that there was the most bone loss in Pifithrin-α group and the least bone resorption in Nutlin-3a group (Fig. 5b, c).

Next, the expression of inflammatory cytokines TNF-α and IL-6 was significantly higher in the periodontal tissues from Pifithrin-α group, but lower in the Nutlin-3a group (Fig. 5d). Additionally, we measured the concentrations of TNF-α and IL-6 in serum of mice peripheral blood, which showed a similar trend with above gene expression results (Fig. 5e).

In order to evaluate macrophages infiltration, immunofluorescence was used to measure the number of M1-type and M2-type macrophages in the mice periodontal tissue between the maxillary first and second molars (Fig. 5f). Compared to periodontitis mice group, Immunofluorescence results showed that the number of M1-type macrophages was increased in Pifithrin-α group, while it decreased in Nutlin-3a group (Fig. 5g). In addition, the number of M2-type macrophages had no significant differences among these groups (Fig. 5h). Obviously, there was a higher ratio of M1/M2 macrophages in Pifithrin-α group and lower ratio of M1-type/M2-type macrophages in Nutlin-3a group (Fig. 5i). These findings suggest that P53 appears to have a greater effect on regulating M1-type macrophages *in vivo*, which was similar with the results *in vitro*.

### Mice with *p53* Deficiency were More Likely to Develop Periodontitis

**Fig. 6:**
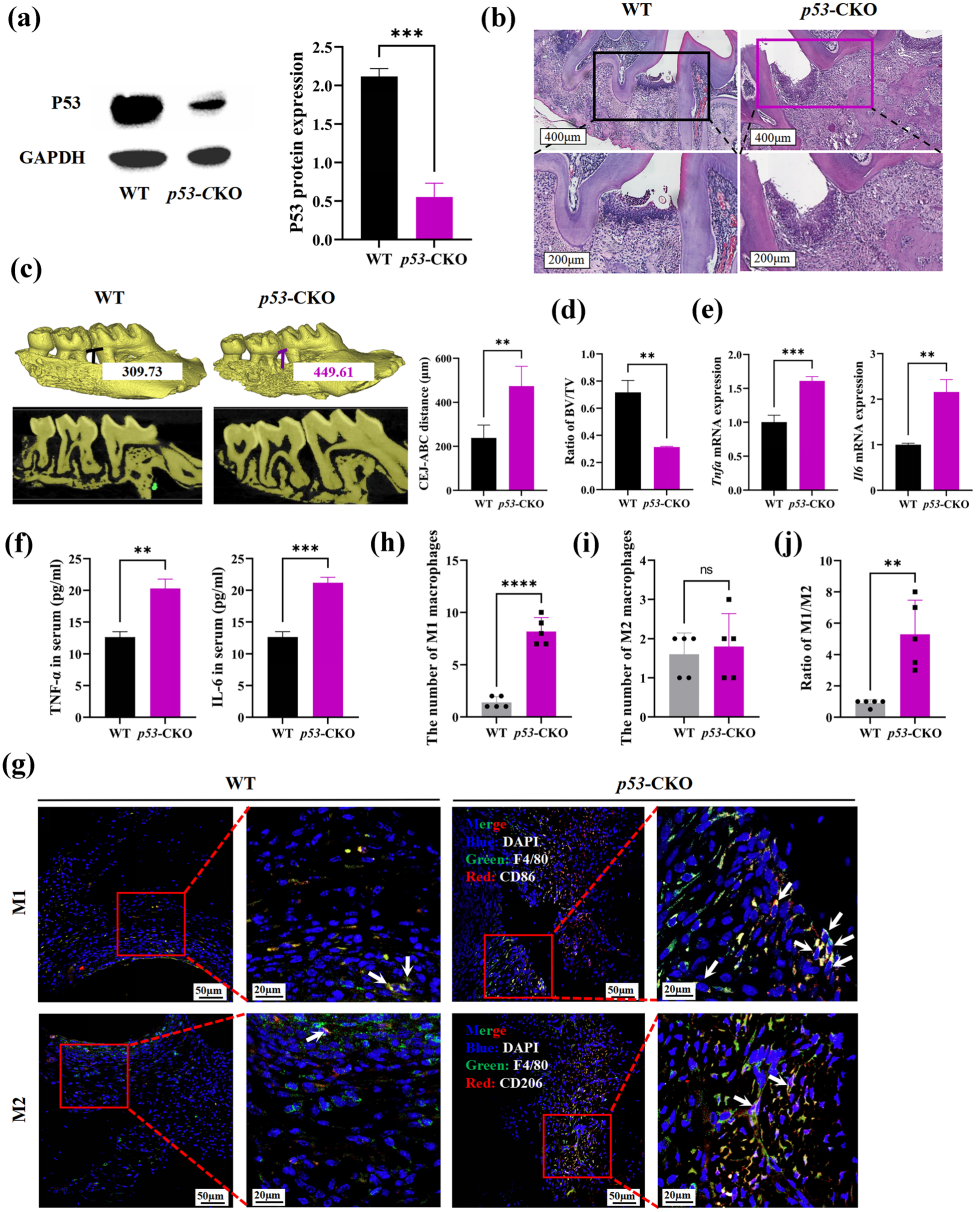
The *p53* gene conditional knockout (*p53*-CKO) mice present more severity periodontitis than wild-type (WT) mice Experimental periodontitis wild-type (*n* = 5) and *p53*-CKO (*n* = 5) mice were established by ligation for 10 days. **a** The expression of P53 protein in periodontitis tissues (*n* = 3). **b** The representative HE staining picture of mice periodontitis tissues in WT and *p53*-CKO group. **c** The absorption degree of alveolar bone in WT and *p53*-CKO group. Representative micro-CT picture was in the left, and the statistics was in the right. **d** The bone mass changes of alveolar bone in each group. **e** The mRNA expression of TNF-α and IL-6 in each group (*n* = 3). **f** The concentration of TNF-α and IL-6 in serum of mice (*n* = 3). **g** The representative immunofluorescence picture of M1-type and M2-type macrophages infiltrated in mice periodontitis tissues. For M1-type macrophages, Blue: DAPI, Green: F4/80, Red: CD86. For M2-type macrophages, Blue: DAPI, Green: F4/80, Red: CD206. The white arrows indicated M1-type or M2-type macrophages. **h**, **i** The number of M1-type macrophages (**h**) and M2-type macrophages (**i**) infiltrated in each group of mice periodontitis tissues. **j** The ratio of M1-type/M2-type macrophages in each group. BV: bone volume, TV: tissue volume, CEJ-ABC: Cemento enamel junction-Alveolar bone crest. ns: no significant, *p < 0.05, **p < 0.01, ***p < 0.001.

To clarify whether the *p53* gene affects the progression of periodontitis through macrophage, *p53*-CKO mice were successfully constructed (Fig. 6a). Compared with wild-type mice, *p53*-CKO mice without any treatment showed no significant change in periodontal tissues (Supplementary Fig. 3C) and bone resorption (Supplementary Fig. 3D). For experimental experiodontitis mice, more subepithelial inflammatory cells infiltration were observed in *p53-*CKO mice than WT mice (Fig. 6b). Micro-CT results showed longer distance from the enamel-dentine boundary to the alveolar ridge (Fig. 6c) and lower alveolar bone mass in *p53-*CKO group (Fig. 6d).

Consistently, we detected higher expression levels of TNF-α and IL-6 in periodontitis tissues (Fig. 6e) and ELISA results showed higher concentration of TNF-α and IL-6 in serum (Fig. 6f). Meanwhile, the M1-type macrophages infiltration and the ratio of M1-type/M2-type macrophages were significantly increased in *p53-*CKO group, but no obvious change for the number of M2-type macrophages (Fig. 6h–j). These results indicated that *p53* conditional knock-out could aggravate the progression of periodontitis by promoting M1-type macrophages infiltration and increasing the expression of inflammatory cytokines.

## DISCUSSION

P53 is known to have significant effects on both inflammation and cancer, but little is currently known about its functional role in the progression of periodontitis. We firstly observed more P53-positive macrophages infiltrated in periodontitis tissues. Next, we found no obvious changes in the differentiation of human and mouse macrophage cells with the treatment of P53 inhibitors or activators alone, but the M1-type polarization of macrophages significantly increased when stimulated by the addition of *Pg.*LPS. Further, higher expression of inflammatory cytokines was also detected in induced cells and cultural supernatants. For *in vivo* study, we established experimental periodontitis mice model by ligation, and found greater alveolar bone loss, higher levels of inflammatory cytokines secretion and more M1-type macrophages infiltration in the application of P53 inhibitors, but the severity of periodontitis was partially alleviated in the application of P53 activators. Notably, to clarify whether the *p53* gene influences periodontitis by regulating macrophage differentiation, we observed more severe periodontitis and M1-type macrophages infiltration in *p53*-CKO mice than in wild-type mice. These findings indicate that the P53 activation may relieve the progression of periodontitis by inhibiting macrophage polarization to M1-type.

As the primary defense mechanism, macrophages are induced to M1-type when combating microorganisms [24, 25]. However, in some diseases, such as lung injury, reducing macrophage accumulation can be beneficial for symptom relief [23]. In septic lung, matrine prophylaxis was found to attenuate the infiltration of M1 macrophages through the SIRT1/P53 pathways, resulting in a decrease in the M1/M2 macrophage ratio [23]. Similarly, an imbalance between inflammatory and reparative mechanisms was observed in local inflammatory periodontal tissue, as evidenced by a significantly higher ratio of M1/M2 macrophages [26]. In human periodontal tissue, we found a higher number of P53-positive macrophages infiltrated in periodontitis groups. Inhibition of P53 induced macrophage polarization toward M1-type, while activation of P53 inhibited macrophage polarization to M1-type. Therefore, regulating P53 may be critical for controlling periodontal disease by maintaining local immune cell homeostasis in the periodontium.

P53 plays a crucial role in both innate and adaptive immune cells [27, 28]. The anti-inflammatory mechanisms of P53 manifest in various ways, with its primary effect being the inhibition of the inflammatory transcription factor NF-κB [11, 29]. Accumulating evidence suggests that NF-κB is associated with M1 macrophage activation [30]. M1 macrophages predominantly secrete pro-inflammatory factors, including TNF-α, IL-6, and IL-1β, while M2 macrophages secrete anti-inflammatory factors such as IL-10 and Arg-1 [20, 21]. Our *in vitro* and *in vivo* experimental results demonstrated that inhibiting P53 increased the expression of TNF-α and IL-6, while activating *p53* decreased their expression, which was consistent with previous studies [31]. Therefore, we suggest that the effect of P53 on the secretion of inflammatory cytokines may be related to the polarization of macrophages.

*In vivo* studies are crucial for overcoming the limitations of *in vitro* studies, as they more accurately replicate the local immune microenvironment of periodontitis and can validate the effects of P53 on disease progression beyond its impact on isolated macrophages. Our results demonstrate that treatment with P53 activator led to a significant improvement in clinical symptoms of mice periodontitis, including reduced alveolar bone resorption and attachment loss, as well as lower levels of inflammatory cytokines in the local tissues and serum. These findings support previous studies that suggest the modulation of macrophage polarization could be a promising strategy for preventing or treating periodontitis in susceptible individuals [32]. Our experimental results indicate that the activation of P53 helps to maintain a healthy M1/M2 macrophage ratio in inflamed periodontal tissues, primarily by inhibiting the differentiation of macrophages into the M1-type. These results suggest that regulating *p53* could be a viable therapeutic strategy for managing periodontitis.

To avoid compensatory mechanisms that may arise from P53 inhibitor administration, we utilized *p53*-CKO mice to construct the periodontitis model and investigate the effects of *p53* deletion on disease progression. Our findings are consistent with those of Claudia Morganti *et al*., supporting the notion that P53 plays a crucial role in regulating the immune response in periodontitis [31, 33]. Our results revealed that *p53*-CKO mice had a more rapid progression and more severe symptoms of periodontitis compared to WT mice, indicating that *p53* deficiency renders mice more susceptible and reactive to periodontitis stimuli. This suggests that P53 plays a crucial role in maintaining the homeostasis of the periodontal immune microenvironment and enhancing resistance to external stimuli. Interestingly, previous study had demonstrated that *p53*-deficient macrophages exhibit increased survival rates of Mycobacterium tuberculosis, whereas the application of P53 activators can decrease this survival rate by regulating the polarization of M1 macrophages [22]. Therefore, P53 activators may have potential therapeutic applications for the treatment of tuberculosis [22]. Given that periodontitis is a chronic infectious disease, we postulate that P53 activation may alleviate periodontitis by reducing the survival rate of *P. gingivalis*, which is the primary pathogen responsible for the disease.

## CONCLUSIONS

Our *in vitro* and *in vivo* experiments demonstrated that P53 has a significant influence on macrophage polarization under inflammatory conditions. Specifically, inhibition of P53 promoted macrophage differentiation towards M1-type, which accelerated the progression of periodontitis. While activation of P53 impaired the ability of macrophages polarization towards M1-type and relieved periodontitis symptoms. Therefore, P53 may represent a potential therapeutic target for managing periodontitis, providing a deeper understanding of the disease mechanisms.

### Supplementary Information

Below is the link to the electronic supplementary material.

Supplementary file1 (PDF 6095 KB)

## Data Availability

The data that support the findings of this study are available from the corresponding author upon reasonable request.
