# Supplementary material for: P53 Alleviates the Progression of Periodontitis by Reducing M1-type Macrophage Differentiation
Source: Inflammation. 2024 Feb 6;47(4):1170–84. doi: 10.1007/s10753-024-01968-w (PMC11343802; doi:10.1007/s10753-024-01968-w)
Supplement: Supplementary file 1 — Supplementary file1 (PDF 6095 KB) [file 10753_2024_1968_MOESM1_ESM.pdf]

P53 alleviates the progression of periodontitis by reducing M1-type macrophage differentiation

Tingting Liu[†1], Dongru Chen[†1], Shanshan Tang[†1], Zhaolei Zou[1], Fangyi Yang[1], Yutian Zhang[1], Dikan Wang[1], Huanzi Lu[1], Guiqing Liao[*1], Xiangqi Liu[*1]

1 Hospital of Stomatology, Guanghua School of Stomatology, Sun Yat-Sen University, Guangdong Provincial Key Laboratory of Stomatology, Guangzhou, China.

†: These authors contributed equally to this work.

*: **Corresponding author:**

[*]Guiqing Liao

liaogq@mail.sysu.edu.cn Department of Oral and Maxillofacial Surgery, Hospital of Stomatology, Sun Yat-Sen University, Lingyuanxi Road, No.56, Guangzhou, China, 510055

[*]Xiangqi Liu

liuxq58@mail.sysu.edu.cn Department of Oral and Maxillofacial Surgery, Hospital of Stomatology, Sun Yat-Sen University, Lingyuanxi Road, No.56, Guangzhou, China, 510055

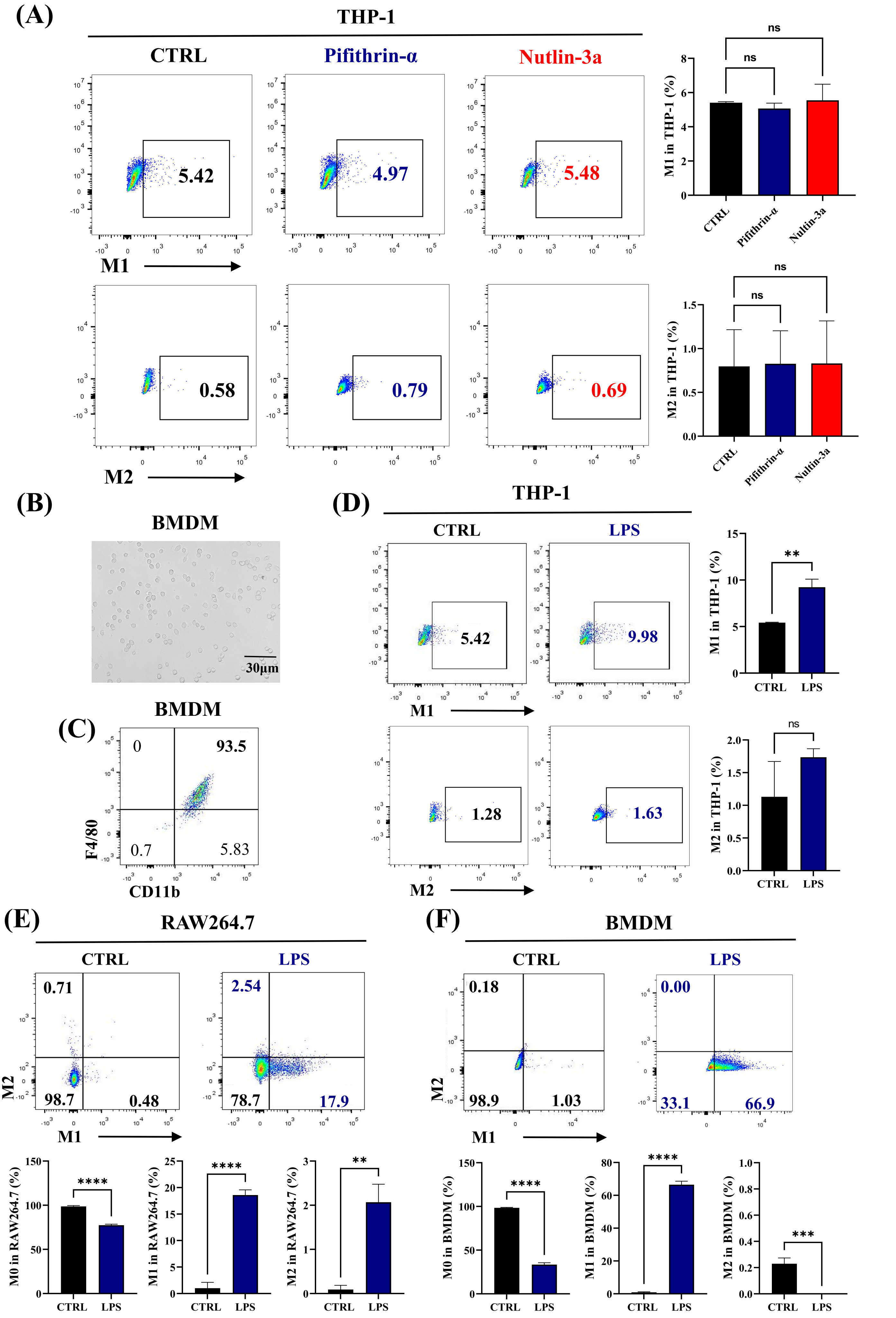

(A)
THP-1
CTRL
Pifithrin-α
Nutlin-3a
5.42
4.97
5.48
M1
M1 in THP-1 (%)
ns
0.58
0.79
0.69
M2
M2 in THP-1 (%)
(B)
BMDM
30μm
(C)
BMDM
0
93.5
0.7
5.83
F4/80
CD11b
(D)
THP-1
CTRL
LPS
5.42
9.98
M1
M1 in THP-1 (%)
**
1.28
1.63
M2
M2 in THP-1 (%)
ns
(E)
RAW264.7
CTRL
LPS
0.71
98.7
0.48
2.54
78.7
17.9
M2
M1
M0 in RAW264.7 (%)
M1 in RAW264.7 (%)
M2 in RAW264.7 (%)
****
**
(F)
BMDM
CTRL
LPS
0.18
98.9
1.03
0.00
33.1
66.9
M2
M1
M0 in BMDM (%)
M1 in BMDM (%)
M2 in BMDM (%)
****
***

Supplementary Fig.1

(A) The proportion of M1 and M2 macrophages in THP-1 cells. THP-1 cells (7×10^5 cells/well) were seeded in a six-wells plate and stimulated with PMA (100ng/mL) for 48 hours, then the cells were cultured in the presence of 20 μM Pifithrin-α or 10 μM Nutlin-3a for 24h. (B) The morphology of BMDM cells cultured with 35 ng/mL recombinant M-CSF for 5 days. (C) The induced efficiency of macrophages from BMDM cells cultured with recombinant M-CSF. (D) The proportion of M1 and M2 macrophages in THP-1 cells (pretreated with PMA) with or without *Pg.*LPS stimulation for 24h. (E, F) Loading RAW264.7 cells (4×10^5 cells/well) and BMDM cells treated with M-CSF (6×10^5cells/well) in a six-wells plate for 24h, then the cells were stimulated by *Pg.*LPS (1μg /mL) for 24h. (E) The proportion of M0, M1 and M2 macrophages in stimulated RAW264.7 cells. (F) The proportion of M0, M1 and M2 macrophages in stimulated BMDM cells. PMA: polymethyl acrylate, M-CSF: macrophage colony-stimulating factor, LPS: lipopolysaccharide. CTRL: control, ns: no significant, $**p<0.01$, $****p<0.0001$.

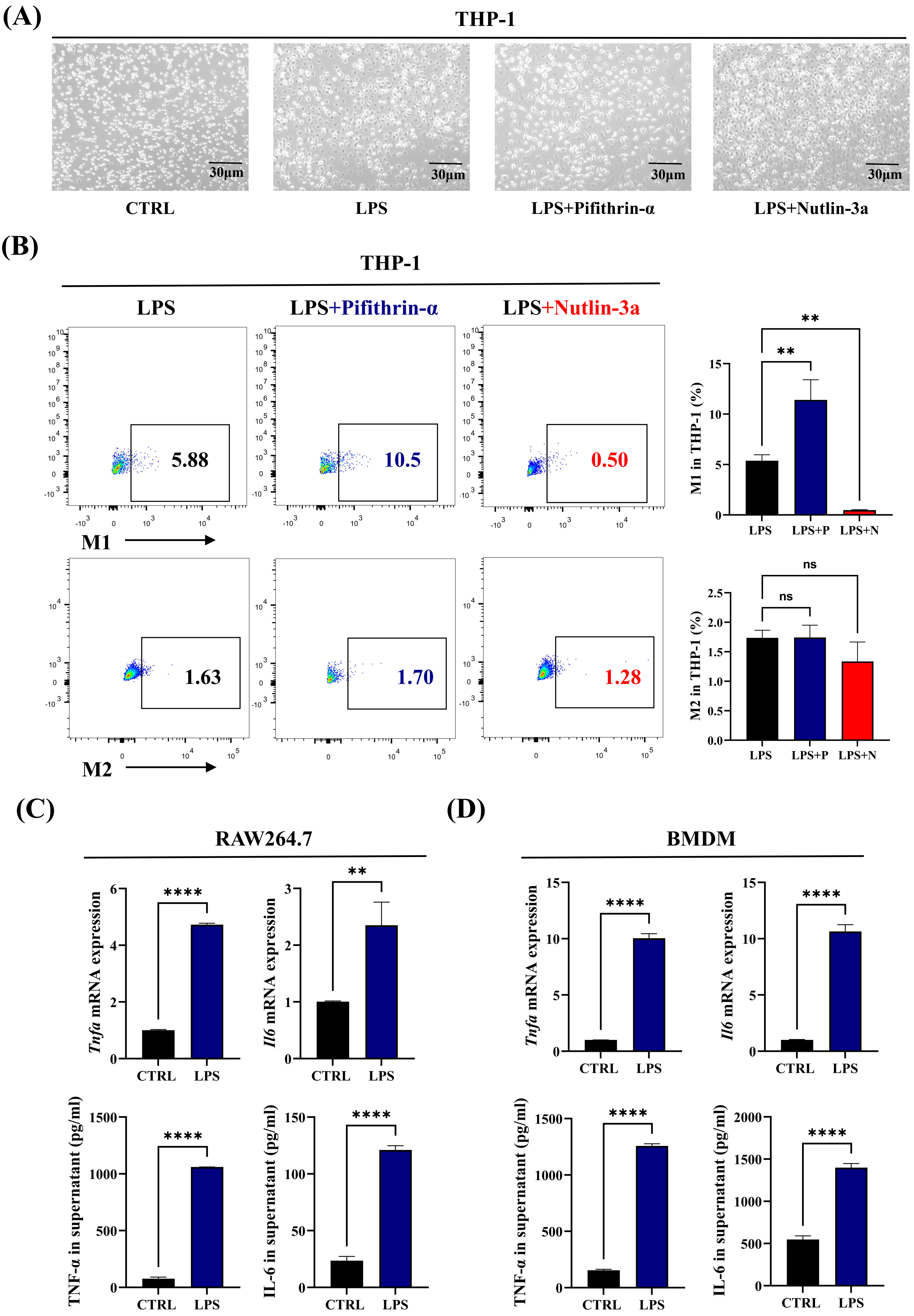
(A)
THP-1
30μm
CTRL
LPS
LPS+Pifithrin-α
LPS+Nutlin-3a
(B)
THP-1
LPS
LPS+Pifithrin-α
LPS+Nutlin-3a
5.88
10.5
0.50
M1
1.63
1.70
1.28
M2
M1 in THP-1 (%)
M2 in THP-1 (%)
**
ns
LPS
LPS+P
LPS+N
(C)
RAW264.7
Tnfa mRNA expression
Il6 mRNA expression
TNF-α in supernatant (pg/ml)
IL-6 in supernatant (pg/ml)
CTRL
LPS
****
**
(D)
BMDM
Tnfa mRNA expression
Il6 mRNA expression
TNF-α in supernatant (pg/ml)
IL-6 in supernatant (pg/ml)
CTRL
LPS
****

Supplementary Fig.2

(A) The cell morphology of each THP-1 cells treated group. THP-1 cells (7×10^5 cells/well) were stimulated with PMA (100ng/mL) for 48 hours and cultured with 20 μM Pifithrin-α or 10 μM Nutlin-3a for another 24h. Next, *Pg.*LPS (1μg /mL) was added for 24h. (B) The proportion of M1 and M2 macrophages in THP-1 cells. (C) The mRNA expression of TNF-α and IL-6 in RAW264.7 cells, and the secretion of TNF-α and IL-6 in cultured supernatant of RAW264.7 cells. (D) The mRNA expression of TNF-α and IL-6 in BMDM cells, and the secretion of TNF-α and IL-6 in cultured supernatant of BMDM cells. LPS: lipopolysaccharide. CTRL: control, ns: no significant, ***p*<0.01, *****p*<0.0001.

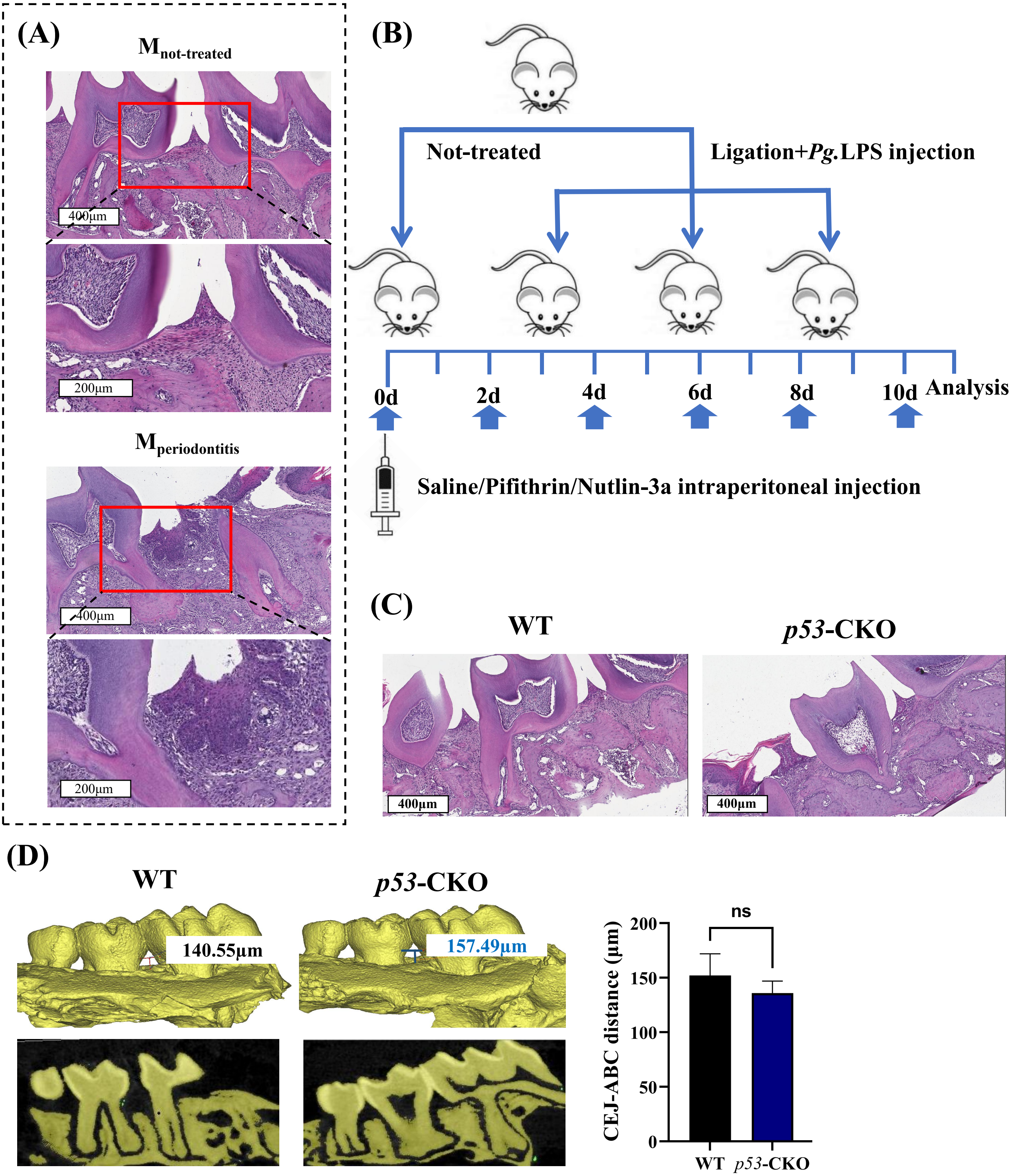
(A)
M not-treated
400µm
200µm
M periodontitis
400µm
200µm
(B)
Not-treated
Ligation+Pg.LPS injection
0d
2d
4d
6d
8d
10d
Analysis
Saline/Pifithrin/Nutlin-3a intraperitoneal injection
(C)
WT
p53-CKO
400µm
400µm
(D)
WT
p53-CKO
140.55µm
157.49µm
CEJ-ABC distance (µm)
ns
200
150
100
50
0
WT
p53-CKO

Supplementary Fig.3

(A) The representative HE staining picture of mice periodontal tissues in control group and Ligation group. (B) The flow chart of the establishment and administration of experimental periodontitis mice model. (C) The representative HE staining picture of mice periodontitis tissues. (D) The comparison of bone resorption between WT and *p53*-CKO mice in physiological status. WT: wild-type, CEJ-ABC: Cemento-enamel junction-Alveolar bone crest, ns: no significant.

Supplementary Table

The primer sequences used in this experiment.

Specie	gene	Sequence 5′-3′	Bp	Tm [°C]
mouse	*Tnfa*	F:CCAGGCAGGTTCTGTCCCTT	20	61.78
		R:ATAGGCACCGCCTGGAGTTC	20	61.97
	Il6	F:CTGGAGCCCACCAAGAACGA	20	62.11
		R:GCCTCCGACTTGTGAAGTGGT	21	62.58
	Gapdh	F：ATCACTGCCACCCAGAAGAC	20	59.67
		R：TGCAAGTGAGCTTCCCGTTC	20	60.88
Human	*TNFA*	F：GAGTGACAAGCCTGTAGCCCA	21	62.05
		R：AGCTCCACGCCATTGGC	17	60.42
	IL6	F：TGTTGGTTGGCAGAGCTGTT	20	60.40
		R：CACCTAAAACACGGCTTGGC	20	60.04
	P53	F：TCCTCCCCAACATCTTATCC	20	55.98
		R：GCACAAACACGAACCTCAAA	20	57.45
	GAPDH	F：GAAGGTGAAGGTCGGAGT	18	55.83
		R：GAAGATGGTGATGGGATTTC	20	53.72
